# Supplementary material for: Transient Topographical Dynamics of the Electroencephalogram Predict Brain Connectivity and Behavioural Responsiveness During Drowsiness
Source: Brain Topogr. 2018 Nov 29;32(2):315–31. doi: 10.1007/s10548-018-0689-9 (PMC6373294; doi:10.1007/s10548-018-0689-9)
Supplement: Supplementary file 1 — Supplementary material 1 (DOCX 4558 KB) [file 10548_2018_689_MOESM1_ESM.docx]

# Supplementary Material


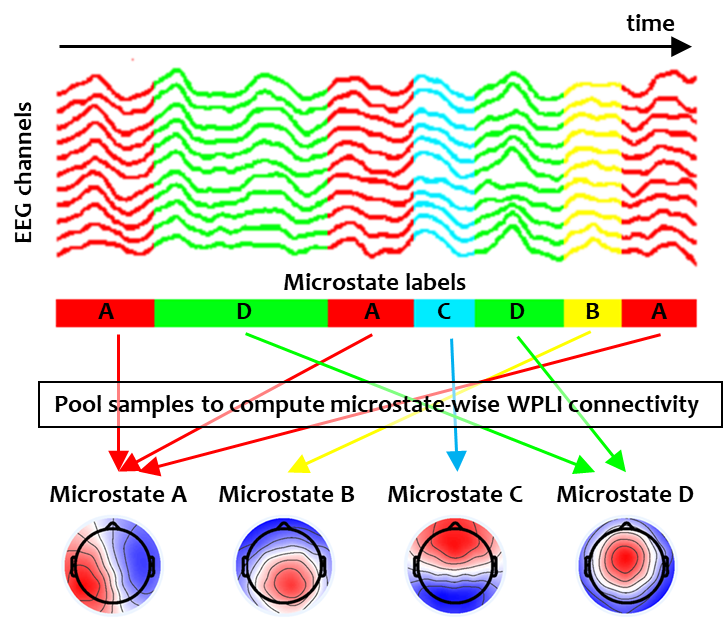


**Supplementary Figure 1. Computation of microstate-wise WPLI connectivity.** Instantaneous channel-wise Hilbert estimates of phase angle at each time sample were pooled together according to the microstate label assigned to the sample. For each microstate, the pooled Hilbert phase angles at each channel were then used to compute WPLI between pairs of channels.


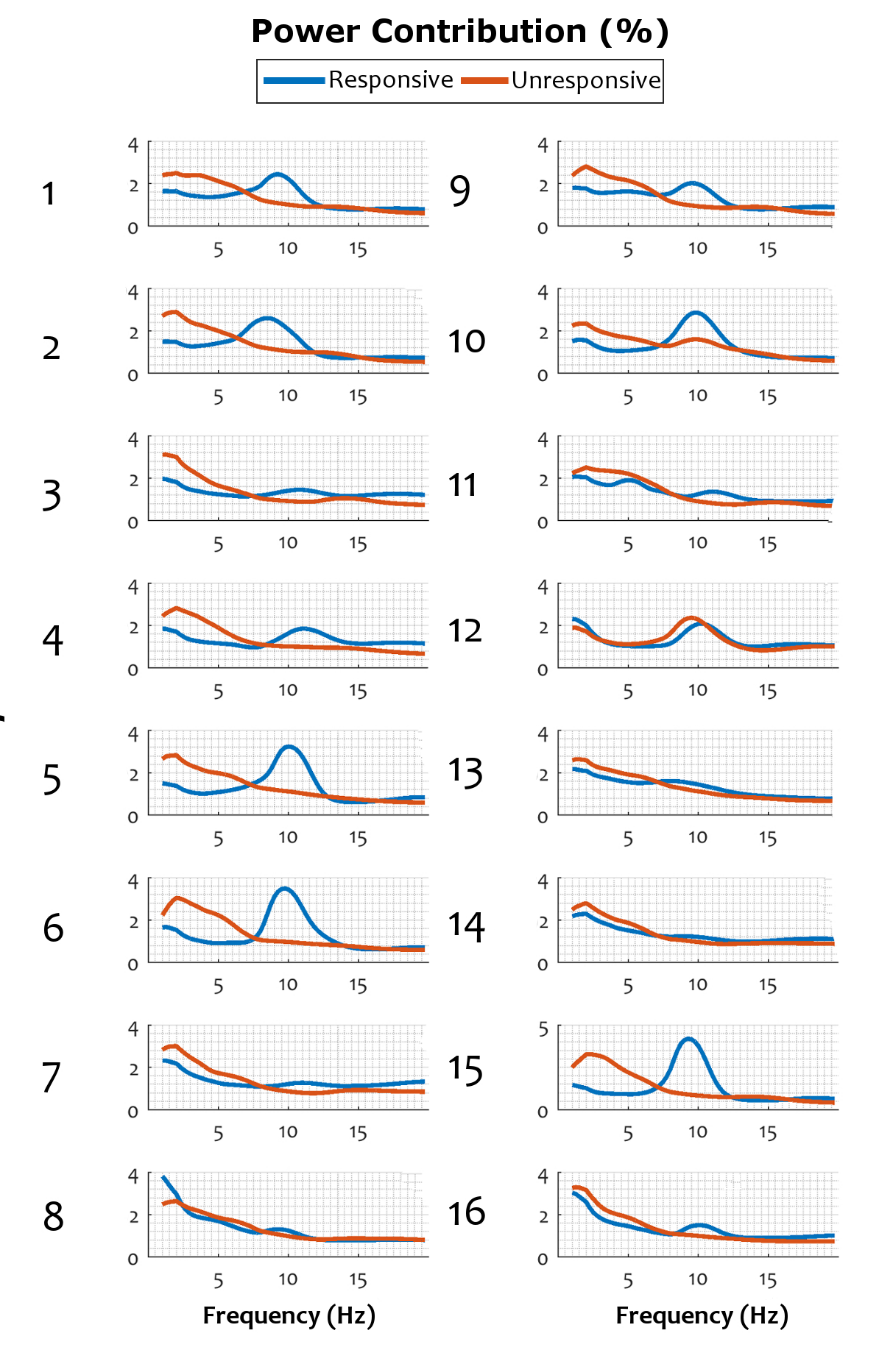


**Supplementary Figure 2. Individual subject spectral power contributions before and after loss of responsiveness.** For each subject, values are averaged over posterior channels (see main text).


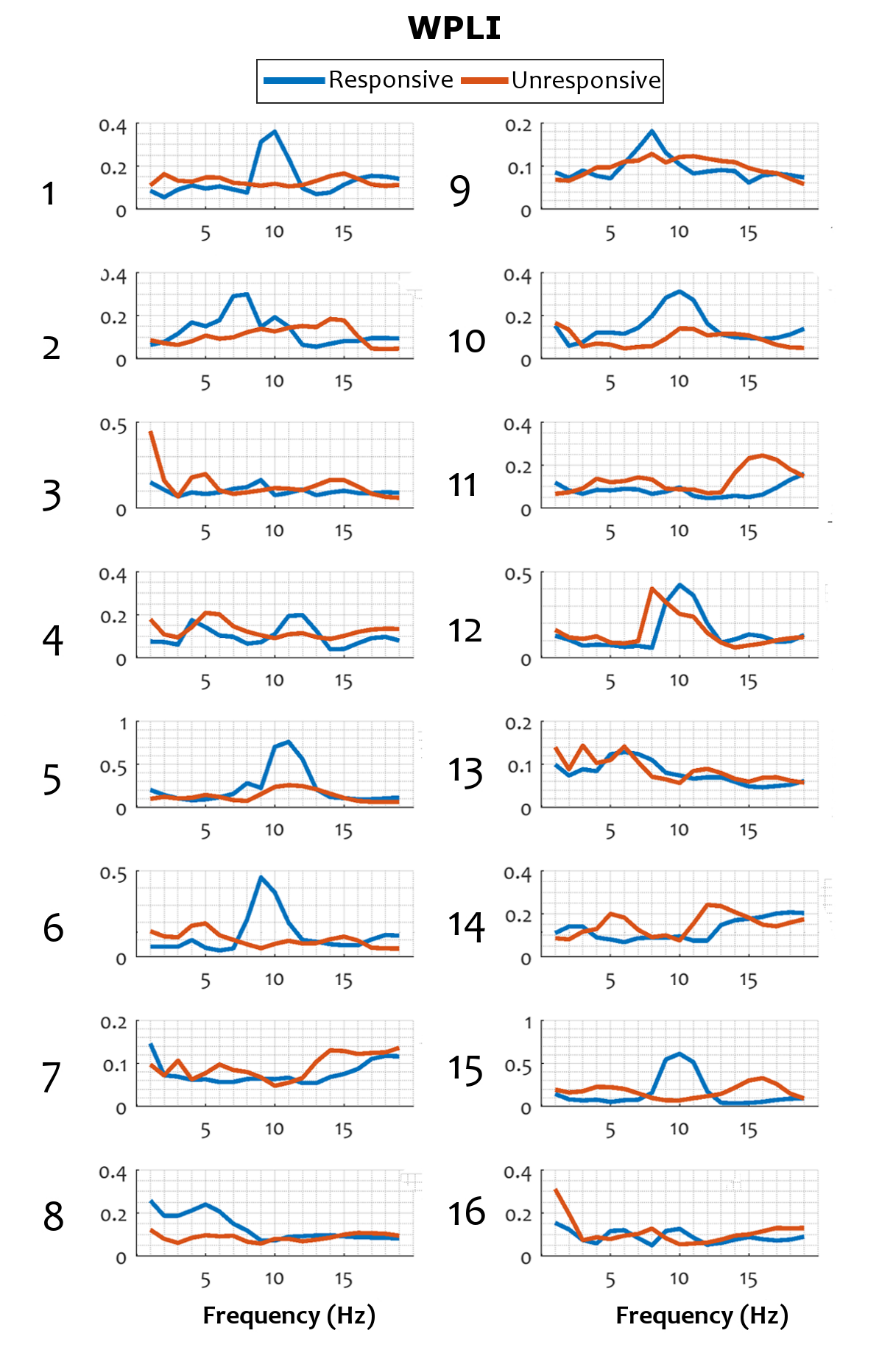


**Supplementary Figure 3. Median WPLI before and after loss of responsiveness in individual subjects.** WPLI values are averaged across all channel pairs.


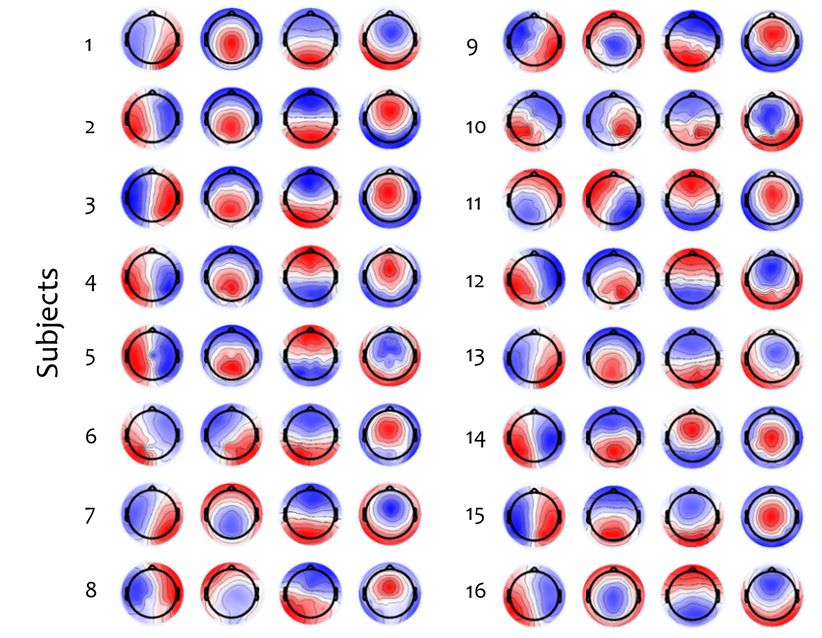


**Supplementary Figure 4. Microstate topographies in each subject, computed over the responsive and unresponsive periods**. In each subject, maps are ordered such that they have maximal spatial correlation with corresponding group-level maps in Fig. 3, ignoring map polarity.

| 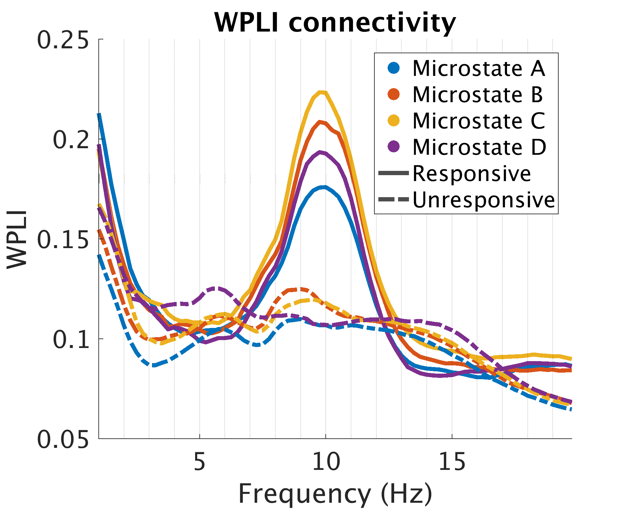  **A** | 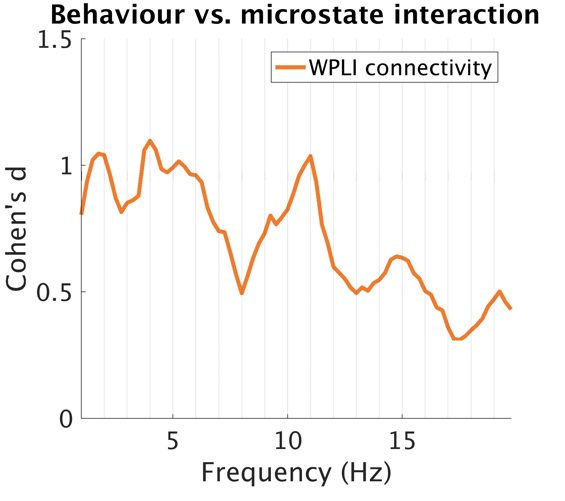  **B** |
| --- | --- |

**Supplementary Figure 5. WPLI connectivity and interaction effect size after Current Source Density Estimation**. Panels A and B re-plot Figs. 6B and 6C after re-estimating WPLI calculated with current source density estimates.
